# Genetic Diversity and Phylogenetic Analysis of *Zygophyllum loczyi* in Northwest China’s Deserts Based on the Resequencing of the Genome

**DOI:** 10.3390/genes14122152

**Published:** 2023-11-28

**Authors:** Mengmeng Wei, Jingdian Liu, Suoming Wang, Xiyong Wang, Haisuang Liu, Qing Ma, Jiancheng Wang, Wei Shi

**Affiliations:** 1 State Key Laboratory of Desert and Oasis Ecology, Key Laboratory of Ecological Safety and Sustainable, Development in Arid Lands, Xinjiang Institute of Ecology and Geography, Urumqi 830011, China; weimengmeng21@mails.ucas.ac.cn (M.W.); ariiiiiink@gmail.com (J.L.); wangxy@ms.xjb.ac.cn (X.W.); www-1256@ms.xjb.ac.cn (J.W.); 2 University of Chinese Academy of Sciences, Beijing 100049, China; 3 College of Forestry and Landscape Architecture, Xinjiang Agricultural University, Urumqi 830052, China; 4 State Key Laboratory of Herbage Improvement and Grassland Agro-Ecosystems, College of Pastoral Agriculture Science and Technology, Lanzhou University, Lanzhou 730020, China; smwang@lzu.edu.cn (S.W.); liuhsh19@lzu.edu.cn (H.L.); maq@lzu.edu.cn (Q.M.); 5 Turpan Eremophytes Botanic Garden, The Chinese Academy of Sciences, Turpan 838008, China

**Keywords:** deserts, China, resequencing, genetic diversity, phylogeny, *Zygophyllum loczyi*, Zygophyllaceae

## Abstract

In order to study the genetics of local adaptation in all main deserts of northwest China, whole genomes of 169 individuals were resequenced, which covers 20 populations of *Zygophyllum loczyi* (Zygophyllales: Zygophylaceae). We describe more than 15 million single nucleotide polymorphisms and numerous InDels. The expected heterozygosity and PIC values associated with local adaptation varied significantly across biogeographic regions. Variation in environmental factors contributes largely to the population genetic structure of *Z. loczyi*. Bayesian analysis performed with STRUCTURE defined four genetic clusters, while the results of principle component analysis were similar. Our results shows that the Qaidam Desert group appears to be diverging into two branches characterized by significant geographic separation and gene flow with two neighboring deserts. Geological data assume that it is possible that the Taklamakan Desert was the original distribution site, and *Z. loczyi* could have migrated later on and expanded within other desert areas. The above findings provide insights into the processes involved in biogeography, phylogeny, and differentiation within the northwest deserts of China.

## 1. Introduction

Widely acknowledged as a type of microevolutionary phenomenon, environmental adaptation means the progressive transformation of organisms across generations [1,2]. Differential selection pressures caused by the spatial heterogeneity of the environment on natural populations may cause a species to adapt variably throughout its range [3,4]. While microevolutionary investigations pertaining to this subject are not uncommon, they are frequently carried out on model plants and cash commodities [5,6,7,8]. A growing number of interests has been focused on the environmental adaptability benefits of genomic population genetics research [9]. The environmental adaptation and genomic differentiation of *Agriophyllum squarrosum* were investigated by Ma et al. via simplified genome sequencing technology [10]. Insufficient reference genomes for organisms other than models, in addition to the lack of clarity regarding the most suitable sample preparation methods and analyses for various research inquiries and evolutionary time scales, have caused a delay in the application of genomes to the study of adaptation in wild desert plants [11]. The evolutionary history of wild desert plants and their adaptation to environmental change require more consideration [12,13,14]. Investigations into the population genetics of desert plant differentiation and adaptation not only yield fresh insights into the study of evolution in its natural habitat, but also present a chance to identify stress-resistance genes that may have significant agricultural implications in the face of climate change [15,16].

Based in the mid-latitudes of the heartland of the Eurasian continent, the Northwest Arid Zone of China has undergone substantial plate tectonic processes [17]. The unique topography formed as a result of these geological processes is composed of expansive inland basins interspersed with towering mountain ranges. Desert basins such as the Taklamakan Desert (TKD), Gurbantunggut Desert (GTD), Badanjilin Desert (BJD), Tengger Desert, Kumtage Desert, and Qaidam Desert (QD) are prominent characteristics of this area. These desert basins are separated by towering mountain ranges [18,19]. These deserts share several inherent attributes: arid conditions characterized by infrequent precipitation, a broad annual temperature spectrum that fluctuates between extreme heat and cold, frequent occurrences of winds and sandstorms, and a vegetation community that is sparse and susceptible to damage [20]. Evidence dates back to the early Cretaceous, according to Wu et al. (1995), which suggests that deserts have existed intermittently in China since at least the Pliocene [21]. During the Early Tertiary, the majority of China’s sandy regions received subtropical arid vegetation [22]. However, as a result of its extensive scale and geographical diversity, vegetation formation differed across different locations, and contemporary communities cannot be classified as either exclusively younger nor uniformly ancient [23]. Quaternary desert evolution and formation resulted from the combined effects of Ice Age climate variability and Tibetan Plateau uplift [24,25]. The Junggar flora, predominantly influenced by their Central Asian component, emerged in the Quaternary period [26,27]. Floral diversity in the Tarim Basin experienced significant expansion during the Quaternary, having its origins in the Early Tertiary [28,29,30]. During the Pliocene of the Late Tertiary, a temperate desert emerged in the Qaidam Basin, which underwent further development during the Quaternary [31,32]. During the Quaternary, the desert flora of Alashan underwent significant development, having originated during the Tertiary [33,34]. Populations may experience large-scale replicative gene duplication events when species distributions are negatively impacted by extreme environments [35]. The correlation between environmental stress and polyploidization events is strong, and it has been suggested that polyploidization can enhance organisms’ capacity to swiftly adapt to severe environmental fluctuations [36,37]. Many plant species, including *Zygophyllum loczyi* (Kanitz, 1891) (Zygophyllales: Zygophyllaceae), which has adapted to arid environments, are found in every major desert basin in the region [38]. As a result of combining phylogenetic analysis and population genetic structure, one can discern the sequence of population formation and the mechanisms underlying the dispersal of widespread plants like *Z. loczyi*. This can provide insights into the overarching characteristics of adaptation and dispersal in the arid regions of Northwest China.

*Z. loczyi* is a C4 herbaceous plant with a life history of one to two years [39,40]. With seventeen species, two subspecies, and three varieties found in China, this genus comprises around 150 species throughout the Old World [38,41,42]. The family of Zygophyllaceae is not only widespread but also prevalent in arid and semi-arid regions, particularly deserts with seasonal dryness [41]. *Zygophyllum* species grow in stony residual dune slopes, fixed and semi-fixed sands, dry riverbeds, gravelly inter-dune flats, and steep loess walls. These species are exceptionally adapted to arid conditions and provide essential ecosystem services in arid environments such as deserts and steppes in the Gobi [43,44,45]. *Zygophyllum* serves as a fundamental component in arid environments due to its susceptibility to wind erosion, drought tolerance, salinity tolerance, and the capacity to thrive in infertile soils. [39,46,47]. Research on the genus has so far focused on its molecular systematics and genetic diversity [48,49,50], morph-anatomy [41,50,51,52], seed biology [53,54], and genetic and chemical aspects of adaptation [55]. Different *Z. loczyi* phenotypes have resulted from the distinct climatic characteristics of China’s desert regions, indicating that local adaptation may be extraordinarily beneficial to comprehend when thinking of plant environmental tolerance.

A total of 169 *Z. loczyi* individuals have been sequenced genetically in this investigation, which spanned four significant desert regions in northwestern China. Our analysis focused on comprehending the potential environmental adaptations of the species in relation to its evolutionary lineage and the geological background of the area.

## 2. Materials and Methods

### 2.1. Sampling and DNA Extraction

**Figure 1:**
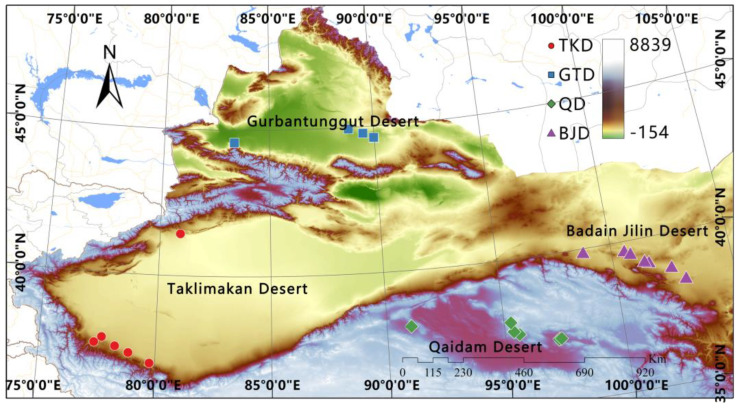
Map of *Z. loczyi* sampling points. Background filled by elevation as color.

A total of 169 plant samples were collected in July 2021 and 2022 from four different desert *Z. loczyi* populations in western China. A total of 28 individuals were from TKD, 35 from GTD, 39 from BJD, and 67 from QD (Table 1). We defined these natural populations as the following four groups: (1) TKD group, (2) GTD group, (3) BJD group, and (4) QD group. Four different regions of *Z. loczyi* seeds were selected to germinate to obtain fresh samples, and the ploidy of each individual was measured by flow cytometry. During sample processing, at least 10 individuals were collected from each population. Detailed records were kept for each sample, including geographic coordinates, elevation, and other environmental conditions at the sampling sites (Figure 1).

### 2.2. Determination of DNA Content by Flow Cytometry

Total genomic DNA was extracted from leaf tissues using the Cetyl Tri-methyl Ammonium Bromide (CTAB) method [56]. The DNA quality and concentration were assessed using 1% agarose gel electrophoresis and a NanoDrop 2000 Spectrophotometer (Thermo Fisher Scientific, Waltham, MA, USA). For resequencing, library construction and sequencing were conducted at Biomarker technologies (Beijing, China) on an Illumina platform (Illumina HiSeq 4000 PE150, Santiago CA, USA), employing a 300-bp read length.

Live samples from different distribution areas of *Z. loczyi* individuals were selected and rinsed repeatedly under running water for 30 s 3–5 times, then dried with tissue paper and set aside. Leaves were digested with both WPB disassociation solution and GLB disassociation solution, respectively, to screen for the suitability of different disassociation solutions. Ploidy was determined using DAPI solution (20 mg/L) staining under UV light during flow cytometry. Genome size was detected using PI solution (20 mg/L) staining and flow cytometry at 632 nm frequency [57,58,59]. We used *Populus tomentosa* (Malpighiales: Salicaceae) leaves as the reference standard.

### 2.3. Genome Resequencing, Assembly, and Annotation

After the evaluation and qualification of the genomic DNA sample, it underwent fragmentation by ultrasound-induced mechanical interruption [60]. The produced fragments were subsequently cleaned by fragment purification, end repair, 3′-end addition of A, connection of sequencing junctions, agarose gel electrophoresis to select fragment size, and PCR amplification to create a sequencing library [61]. Clean Reads were obtained after the Raw Reads were filtered to eliminate those containing adapters, exceeding 10% N content, or more than 50% bases with a quality value below 10 [62].

As the sequencing accuracy escalates in relation to the length of the sequenced reads, the quality values had been transformed into error rates and executed the base type of distribution analysis to detect the existence of AT and GC segregation [63]. Due to the fact that *Z. loczyi* is known as a wild plant and acquiring the reference genome of close relatives has a stronger challenge, *Zygophyllum. Xanthoxylum* is selected by us, which is also a species of the *Zygophyllum* genus, as the reference genome [44]. It is necessary to transfer the clean sequences obtained by sequencing to the reference genome. Therefore, we compared the Clean Reads with the reference genome using bwa-mem2 (v2.2) software, sorted the results using samtools (v1.9) sort comparison, and statistically calculated the sequencing depth and genome coverage of each sample based on the sorted results [64,65].

We determined the starting and ending positions of the reference genome’s double-ended sequence. The CollectInsertSizeMetric.jar application from the Picard (v2.25.5) software toolset is used for calculating the insert fragment’s size subsequent to the interruption of the sample DNA [66,67].

### 2.4. SNP and Variant Detection and Annotation

SnpEff [4] is software made to identify the impact of variants and to annotate variants [68]. To ensure the reliability of SNPs, the statistical cumulative distribution of distances between neighboring SNPs is used along with the number of reads that correlate with the detected SNPs [69]. The finding of the variant locus’s site and the consequence of the variant can be accomplished by utilizing the reference genome’s gene position information combined with the variant locus’s position.

Detection of SNPs and InDels was performed using GATK (v3.8) [70]. To ensure the accuracy of the detection results, redundant reads were filtered using samtools (v1.9) based on the alignment of cleaned reads to the reference genome [64,65]. Subsequently, the GATK HaplotypeCaller algorithm was employed for SNP and InDel variant detection. Through filtering, a final set of variant sites was obtained and stored in VCF format [71]. Using the vcfutils.pl subroutine of bcftools (var Filter-W 5-W 10), SNPs is filtered out SNPs in the 5 bp range of InDels and neighboring InDels in the 10 bp range. Cluster Size is set to 2 and Cluster Window Size to 5, indicating that the number of variants in a 5 bp window should not exceed 2. We filtered out variants with quality scores below 30, QD values below 20, FS values above 60, and/or MQ values below 40. Other variant filtering parameters followed the default values specified by GATK. Making use of the Circos (0.69-9) software, the distribution of the results for each type of mutation obtained from the assay was plotted [72].

The annotations of these genes were accessible for the purpose of analyzing the functions of the genes through the comparison of variant genes with functional databases maintained by Diamond, including NR, Swiss Prot, GO, COG, and KEGG [73,74,75,76,77].

### 2.5. Genetic Evolution Analysis

The population structure and admixture are inferred among our 169 samples using MEGA X (https://www.megasoftware.net/, accessed on 25 July 2023) under the Kimura 2-parameter model; clade support was calculated using 1000 bootstrap replications [78]. We also performed clustering analyses as a complimentary way to detect genetic structure. The population genetic structure of *Z. loczyi* was assessed by employing ADMIXTURE (v1.22) and utilizing high-quality SNPs [79]. The most likely number of clusters was computed with 10-fold cross-validation (CV), comparing K-values from 2 to 10.

A PCA based on SNP using the smartPCA program (https://data.broadinstitute.org/alkesgroup/EIGENSOFT/EIG-6.1.4.tar.gz, accessed on 25 July 2023) in EIGENSOFT also be created (v6.0) (https://www.megasoftware.net/, accessed on 25 July 2023) to study genetic relatedness and clustering among populations [80]. Finally, we created a kinship heat map for estimation of kinship between any two individuals using GCTA (v1.92.1) (https://yanglab.westlake.edu.cn, accessed on 25 July 2023) [81]. The PopLDdecay has been used (v3.41) to estimate linkage disequilibrium (LD) decay based on the coefficient of determination (r^2^) between any two loci (https://github.com/BGI-shenzhen/PopLDdecay, accessed on 25 July 2023) [82]. The Plot_MultiPop.pl script that comes with the software was then used to plot the decay curve.

Diverse population genetics metrics were computed utilizing the VCFtools (0.1.15) software utility, with a sliding window of 100 kb and a step size of 10 kb, the SNPs that exhibited the highest degree of consistency [71].

## 3. Results

### 3.1. Quality Control of Sequencing Data

#### 3.1.1. Genome Size and Sequencing

**Figure 2:**
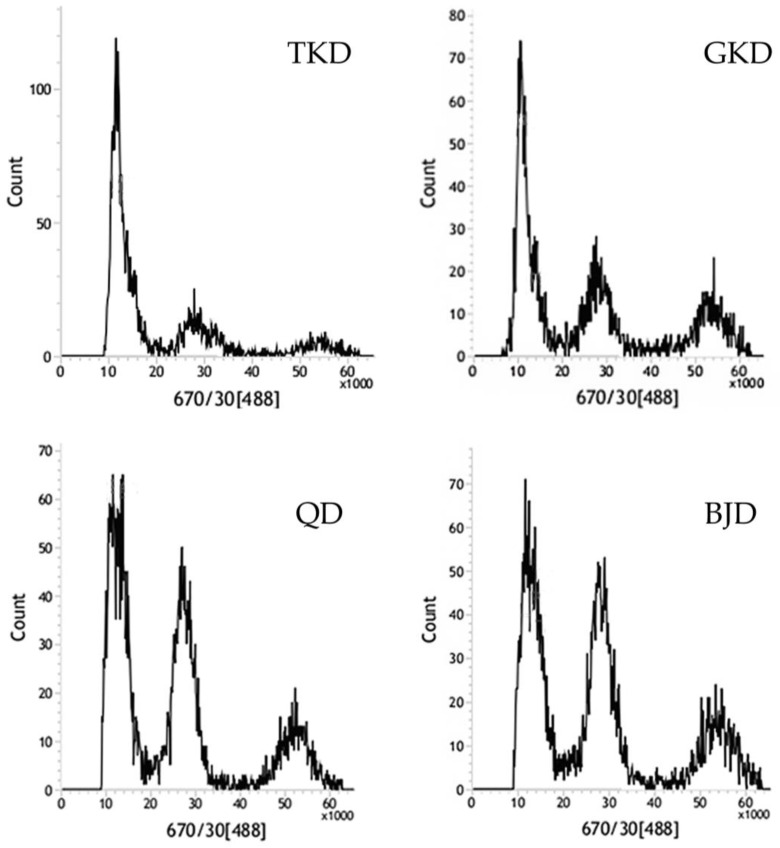
Four different *Z. loczyi* populations’ DNA content and ploidy measured by 670-30A Dual-beam Infrared Spectrophotometer. The excess spectral absorption peaks may be a result of uneven cell staining.

As *Z. loczyi* is a non-model species, we used *Z. xanthoxylum* for a reference genome (NCBI BioProject PRJNA933961). By flow cytometry, we determined that the *Z. loczyi* chromosomal ploidy is diploid, with a genome size of approximately 500 Mb. (Figure 2). A total of 1491.98 Gbp of genome-pure data were obtained by resequencing, with Q30 reaching 91.96–95.75% and an average GC content of 34.28%. The alignment rate between the sample and the reference genome was about 60.77%, while average coverage depth was average 3.81× (Supplementary Table S1).

#### 3.1.2. Analysis of Base Sequencing Quality Distribution

**Figure 3:**
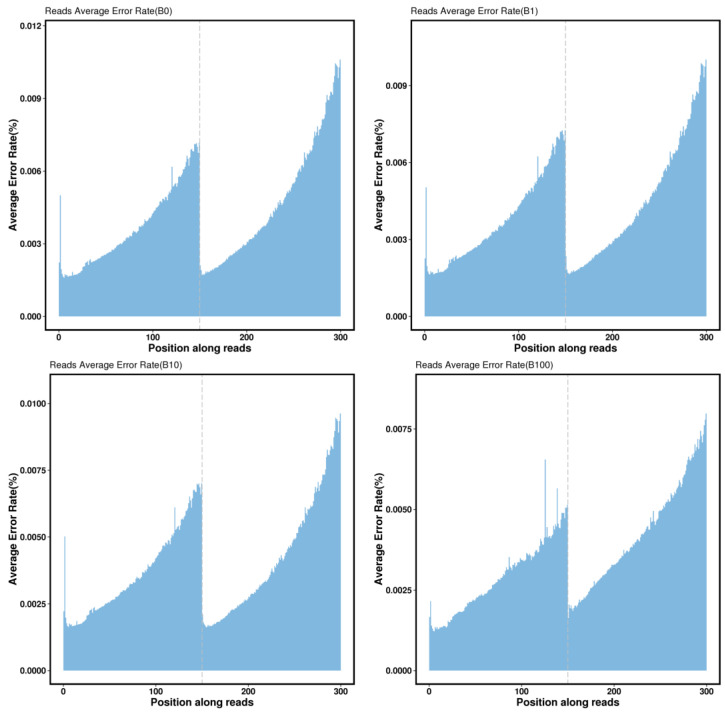
Distribution of base error rate among part of *Z. loczyi* samples. The horizontal coordinate is the base position of the Reads, and the vertical coordinate is the single base error rate. The first 150 bp is the distribution of error rate of the first end of the sequenced Reads of the bipartite sequenced sequence, and the last 150 bp is the distribution of the error rate of the other end of the sequenced Reads.

**Figure 4:**
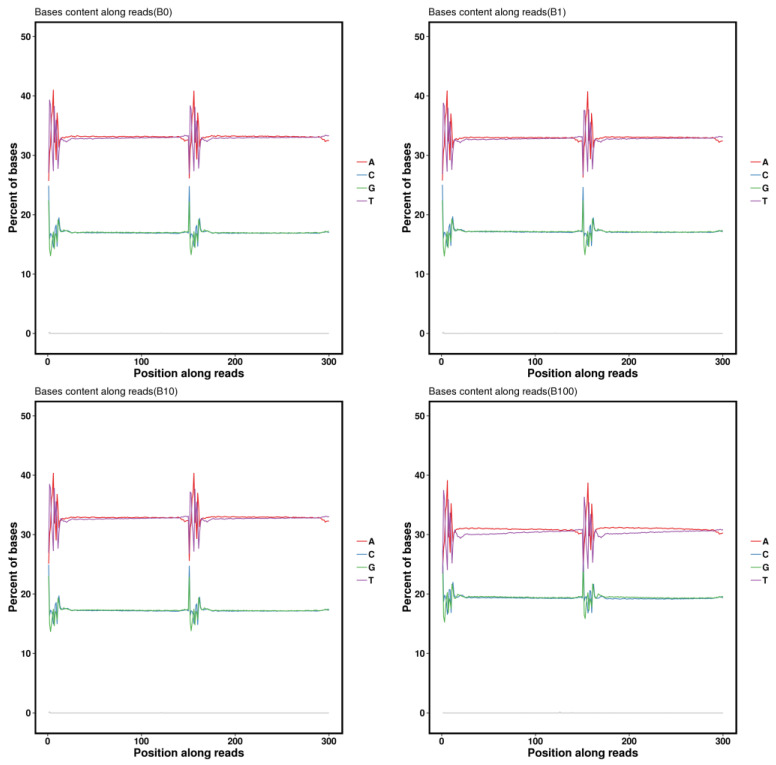
Distribution of the proportion of each base of the bases in some samples of *Z. loczyi*. The horizontal coordinate is the base position of the Reads, and the vertical coordinate is the proportion of bases; green represents base G, blue represents base C, red represents base A, purple represents base T, and grey represents base N that was not identified in sequencing. The first 150 bp is the base distribution of the first end of the sequenced Reads of the bipartite sequencing sequence, and the last 150 bp is the base distribution of the sequenced Reads of the other end of the sequence. The first 150 bp is the base distribution of the first end of the sequenced Reads of the double-ended sequences, and the second 150 bp is the base distribution of the sequenced Reads of the other end.

During the execution of base sequencing quality distribution analysis, it was observed that the samples which include the final dozen bases and the first four bases show lower quality values compared to the intermediate sequencing bases. However, all of these samples carried quality values more than Q30%. To illustrate that, we transformed the quality values into error rates and graphically represented the error rate distribution as follows (Figure 3). The examination of base type distribution showed that AT and CG bases were basically not separated, the curve was gentle, and the sequencing results were normal (Figure 4).

#### 3.1.3. Analysis of Reference Genome Comparisons

Comparison with the reference genome has shown that there is no contamination in the experimental process, and graphing based on the depth of coverage of each chromosome locus shows that the genome is covered more evenly, indicating better sequencing randomness. The uneven depth on the graph may be due to repeated sequences, PCR preference.

By detecting the start and stop positions of the bipartite sequences on the reference genome, the precise measurements of the sequenced fragments acquired subsequent to the interruption of the sample DNA could be ascertained. This analysis confirmed that the length distribution of the insert fragments followed a normal distribution, suggesting that the library construction of the sequencing data was normal.

**Figure 5:**
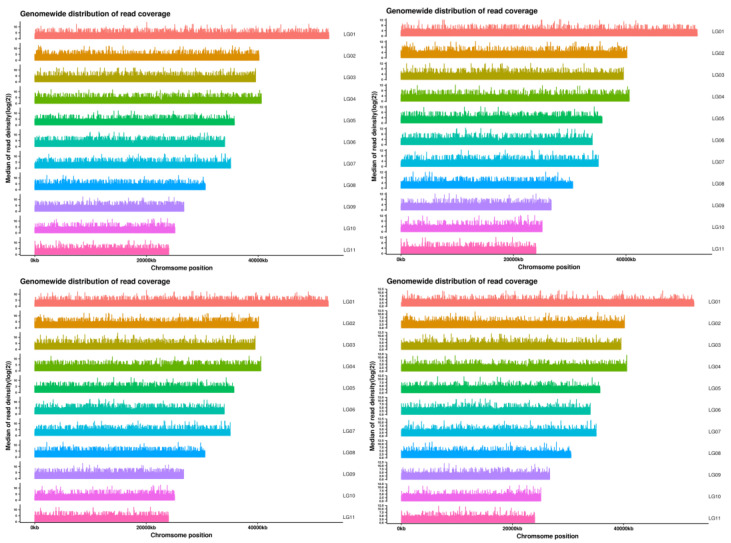
Chromosome coverage depth distribution of some samples of *Z. loczyi*. The horizontal coordinate is the chromosome position and the vertical coordinate is the value obtained by taking the logarithm of the depth of coverage at the corresponding position on the chromosome.

**Figure 6:**
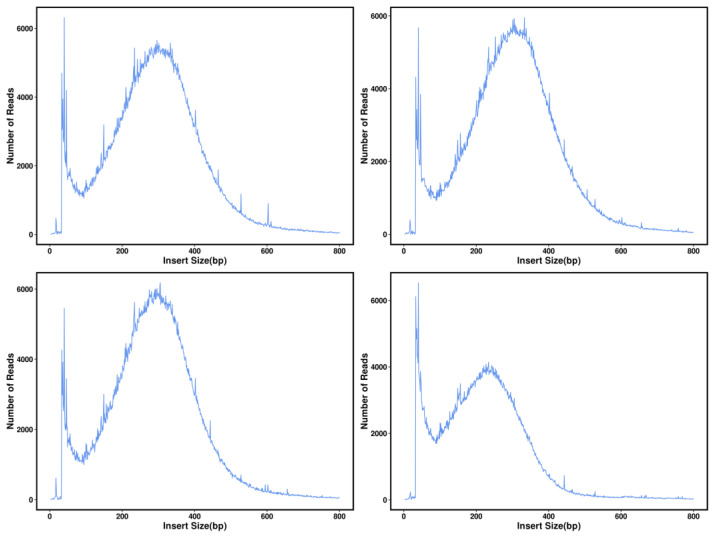
Distribution of *Z. loczyi* insert fragments. The horizontal coordinate is the length of the inserted segment and the vertical coordinate is its corresponding number of Reads.

**Figure 7:**
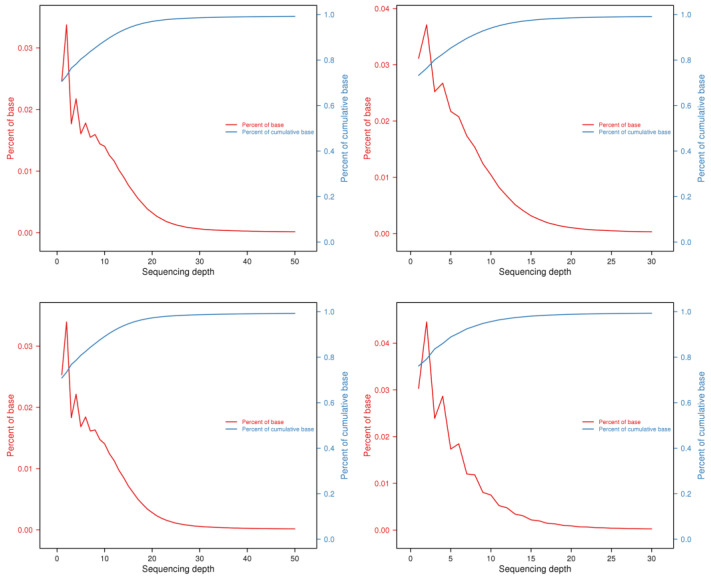
Distribution in depth of a selection of *Z. loczyi* samples. The above figure reflects the basic distribution of sequencing depth, with the horizontal coordinate being the sequencing depth; the left vertical coordinate being the percentage of bases corresponding to that depth, which corresponds to the red curve; and the right vertical coordinate being the percentage of bases at and below that depth, which corresponds to the blue curve.

After localization to the reference genome, the number of Reads can be discovered with the quantification of base coverage on the reference genome (Figure 5). A more uniform distribution of bases on the genome in terms of coverage depth suggests that the sequencing randomness has been enhanced. Figure 6 below illustrates the coverage distribution curve and base coverage depth distribution curve of the samples (Figure 7).

#### 3.1.4. SNP Identification and Quality Control

**Figure 8:**
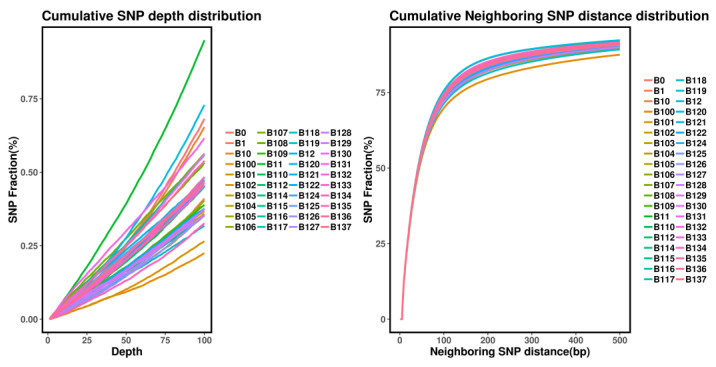
A cumulative plot of the number of SNP Reads supported is shown on the left, and a cumulative plot of the distance between neighboring SNPs is shown on the right.

To provide a genome-wide overview of the dynamics underlying local adaptation, a total of 169 *Z. loczyi* individuals were collected from 20 natural populations across their current distribution in China (Figure 1). Based on these population samples, our genome resequencing approach yielded 232,724,423 high quality SNPs (allele frequency > 0.05 and integrity > 0.8) which were used for subsequent population genetic analyses (Figure 8). To ensure the reliability of the SNPs, we examined the cumulative SNP depth distribution to identify the predominant SNP types and their frequencies. Within the 25–75% interval, the SNPs displayed high depths with pronounced peaks, suggesting that the SNPs are of better quality (Figure 8).

#### 3.1.5. Detection and Distribution of Variation

**Figure 9:**
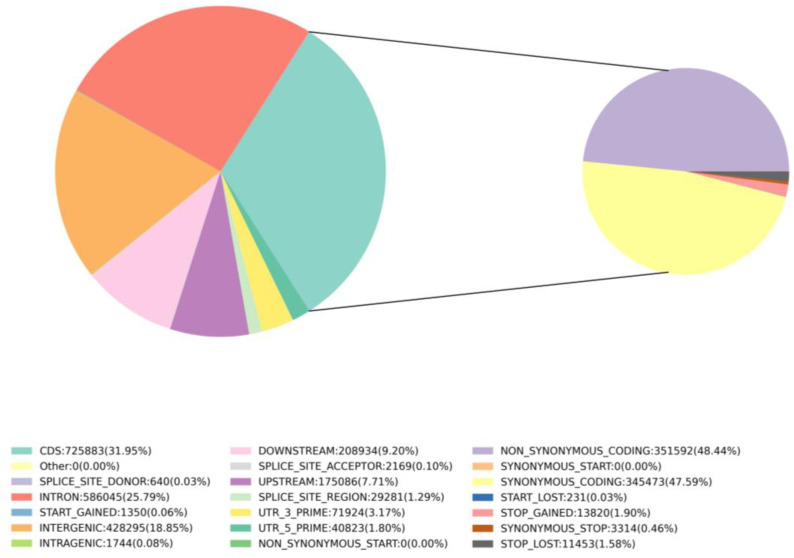
Classification results based on the reference genome SNP annotation of all samples. Proportions of the various SNPs in the *Z. loczyi* and the reference genome of *Z. xanthoxylum*.

A total of 150,819,465 SNPs were detected, with a Het-ratio (heterozygosity/homozygosity) of 0.65% to 2.99%. The Ti/Tv (Transition/Transversion) ratio ranged from 1.38 to 1.43. These values are based on a Ti range of 419,115–607,294 and a Tv range of 295,847–437,912, which correspond to different samples (Supplementary Table S1). A comprehensive analysis of the detected SNPs revealed distinct distribution patterns among different genomic regions. Among all the SNPs identified, 18.85% were classified as intergenic, 25.79% were found in intronic regions, and 31.94% were within CDS (Figure 9). Notably, among the CDS SNPs, a significant proportion consisted of non-synonymous coding variants (15.47%) and synonymous coding variants (15.20%) (Figure 9). These findings highlight the prevalence of genetic variation within protein-coding regions, with potential functional implications associated with both non-synonymous and synonymous alterations.

**Figure 10:**
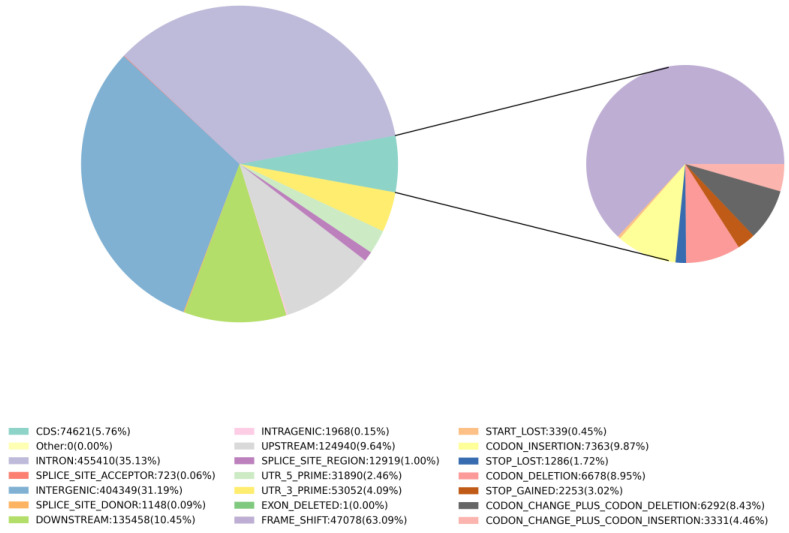
Classification results based on the reference genome InDel annotation of all samples. Proportions of the various InDels in the *Z. loczyi* and the reference genome of *Z. xanthoxylum*.

A total of 1,296,479 InDels were detected in the dataset. The heterozygosity ranged from 2866 to 12,552, while the homozygosity ranged from 360,119 to 701,259. The Het-ratio varied from 0.75% to 2.16% (Supplementary Table S1). In terms of distribution across different genomic regions, introns accounted for 0.35% of the total InDels, intergenic regions represented 0.31%, downstream non-coding regions accounted for 0.10%, upstream non-coding regions represented 0.09%, and the CDS accounted for 0.06% (Figure 10). Within the CDS category, the main subtypes of InDels were frameshifts (0.04%) and codon-insertions (0.006%) (Figure 10). These findings provide insights into the prevalence and distribution of InDels, including within protein-coding regions, suggesting potential functional implications of genetic variation in the studied population.

**Figure 11:**
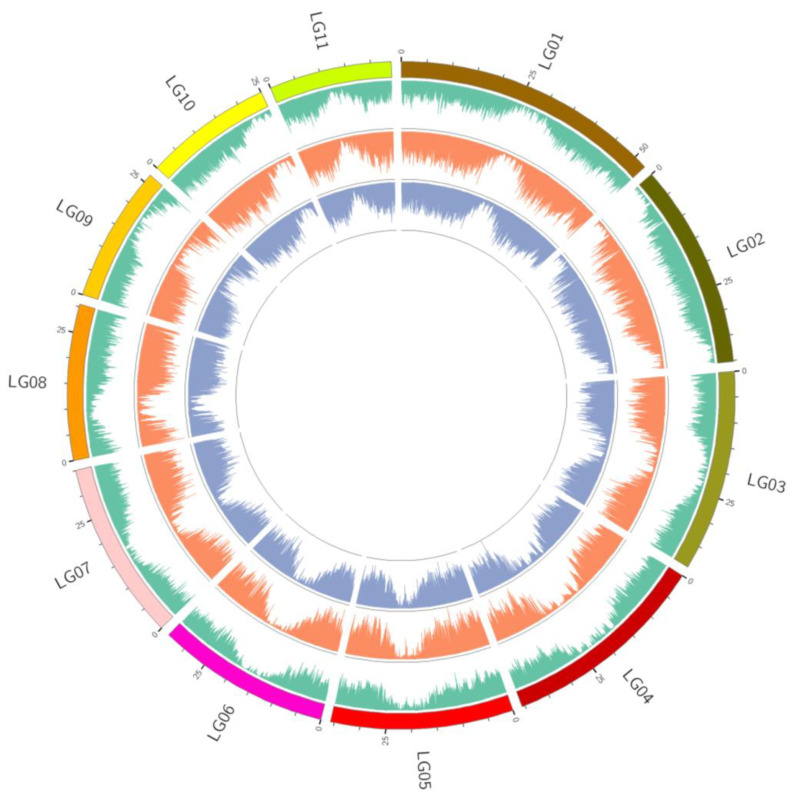
Distribution of SNPs and InDels detected in the *Z. loczyi* and the reference genome of *Z. xanthoxylum*, in the 11 chromosomes (color block = chromosome coordinates, green line = gene density distribution, orange line = SNP density distribution, purple line = InDel density distribution).

The SNP density across various chromosomes is depicted in Figure 11. Chromosome 1 exhibited the highest density of SNPs, with a count of 325,704 SNPs, while chromosome 9 displayed the lowest SNP density, comprising 132,516 SNPs (Figure 11). Within each chromosome, the distribution of polymorphism was uneven, encompassing both densely populated and sparsely populated regions of SNPs.

#### 3.1.6. Genomic Signals of Adaptation

**Figure 12:**
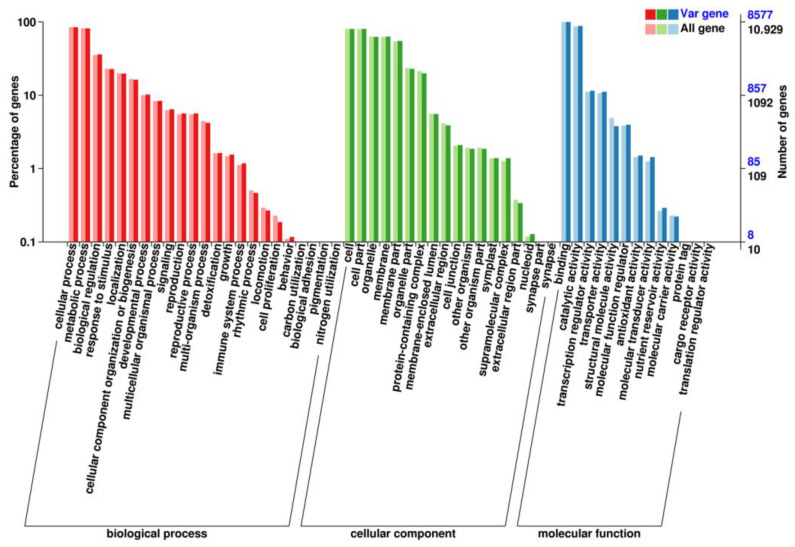
The SNPs of *Z. loczyi* annotation clustering according to the GO.

**Figure 13:**
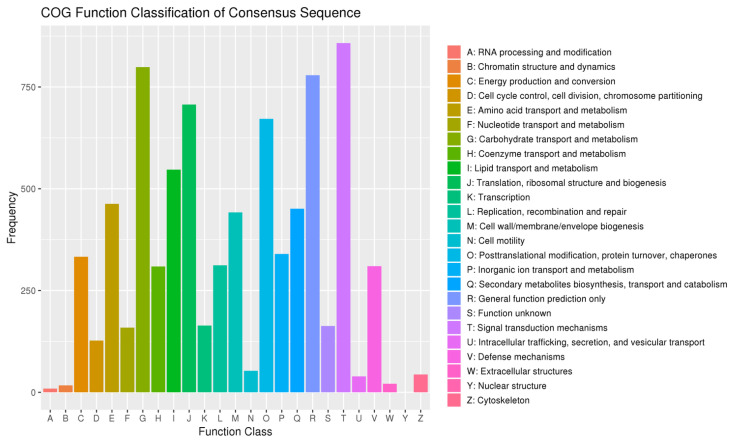
The SNPs of *Z. loczyi* annotated according to the COG database; the *x*-axis shows the taxonomical content of the COG data; the *y*-axis shows the number of genes.

GO analysis was performed to elucidate gene functions across three major categories: biological processes, cellular components, and molecular functions (Figure 12). The GO analysis of biological processes revealed the involvement of genes in various essential biological activities. These processes ranged from fundamental cellular functions such as metabolism, cell cycle regulation, and signal transduction, to more specialized processes like immune response, development, and neuronal signaling. In terms of cellular components, the GO analysis provided insights into the localization and organization of gene products within cells. The variant gene COG categorization statistics revealed that the most prevalent items were T (signal transduction mechanisms), G (carbohydrate transport and metabolism), R (general function prediction only), and J (translation, ribosomal structure, and biogenesis) (Figure 13).

### 3.2. Genetic Evolution Analysis

#### 3.2.1. Genetic Diversity

Based on the population structure of *Z. loczyi*, we calculated seven genetic indices (MAF, Ae, Ao, He, Ho, PIC, and I) for each clade and population. The MAF across the four clusters ranged from 0.25 to 0.28, demonstrating relatively consistent values. The QD clade exhibited the highest genetic diversity (He = 0.365), followed by the TKD clade (He = 0.353) and the BJD clade (He = 0.333), while the GTD clade had the lowest genetic diversity (He = 0.318) (Table 2). These findings suggest that within the other three populations, there exists a non-random distribution of genotypes among individuals, possibly attributable to selection for specific beneficial genotypes or a heterozygote advantage at polymorphic loci. In contrast, the QD population demonstrated the Ho lower than the He, implying a genotype distribution closer to random among individuals in this group, devoid of discernible selective advantages or excess heterozygosity effects.

When using Nei’s diversity index, the mean values for the four groups were as follows: TKD = 0.36, GTD = 0.323, QD = 0.368, and BJD = 0.337. Based on these mean values, the QD group displayed the highest Nei’s diversity, while the GTD group had the lowest. In this study, all populations showed medium variation (0.25 < PIC < 0.5). We also calculated the Shannon Information Index for each of the four populations: TKD (0.523, 0.09–0.693), GTD (0.476, 0.075–0.693), QD (0.540, 0.044–0.693), and BJD (0.498, 0.069–0.69). The TKD group had the highest number of polymorphic markers (45,656), while the BJD group had the lowest number of polymorphic markers (41,169). These findings demonstrate the diversity and complexity of information across these groups. Despite the relatively low average values, the wide distribution suggests the presence of distinct sources of genetic information and unique characteristics within each group.

#### 3.2.2. Phylogenetic and Population Genomic Analyses

**Figure 14:**
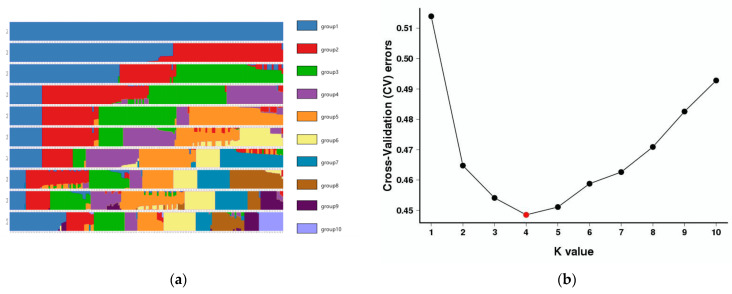
(**a**) Clustering results of samples corresponding to each of the ADMIXTURE population genetic structure; (**b**) Genetic structure analysis of *Z. loczyi* based on the Bayesian model, The red dot represents the appropriate K value.

The optimal ancestral clustering at K = 4 was determined based on the cross-validation error rate (Figure 14). The geographic divisions observed in the population align closely with the actual geographic divisions.

**Figure 15:**
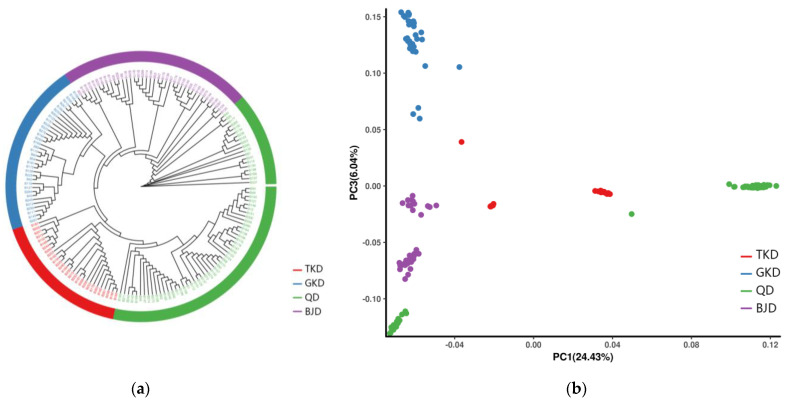
(**a**) Phylogenetic trees were generated for each sample by employing neighbor-joining with 1000 bootstrap replications and the Kimura 2-parameter model.; (**b**) the sample is clustered in two dimensions using principal component analysis (PCA), where PC1 and PC3 denote the first and third principal components, respectively. A color denotes a group, while a dot represents a sample.

We also reconstructed the phylogenetic relationship of the 20 populations based on the same SNP dataset using the neighbor-joining method. The results are generally consistent with the population structure detailed above; however, the QD group is further divided into two subgroups (Figure 15a). Principal component analysis (PCA) further supported the existence of four distinct groups among the 20 populations (Figure 15b). Notably, although *Z. loczyi* exhibited a distinct spatial structure according to various genomic methods, a relatively small amount of genetic variation was observed. Additionally, PCA and ADMIXTURE analyses based on the Bayesian algorithm corroborated the population structure observed in the phylogenetic tree. The optimal clustering solution for the populations was K = 4. Similarities existed in terms of population composition and geographic dispersion.

#### 3.2.3. Linkage Disequilibrium Decay Analysis

**Figure 16:**
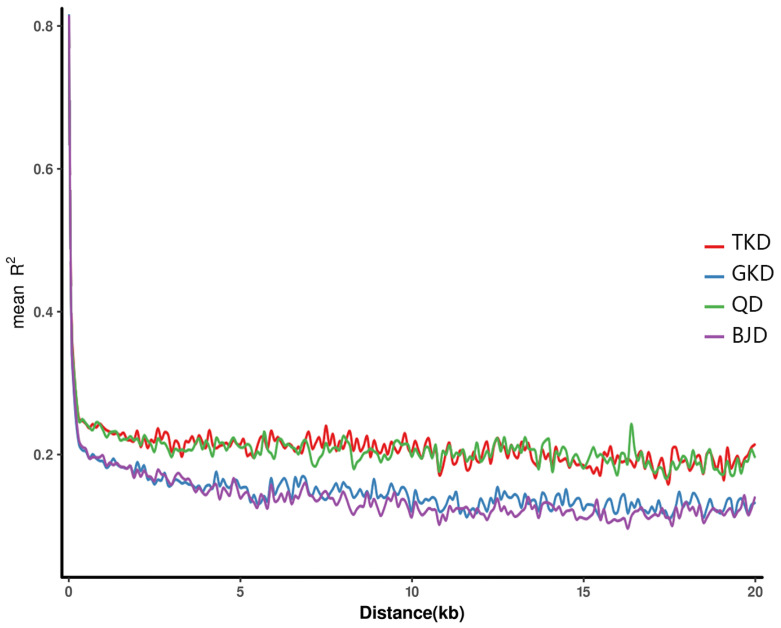
Linkage disequilibrium (LD) is a measure of whether genotypic changes in two molecular markers are in step and correlated.

The LD between any two SNPs within a certain distance range (20 kb) was calculated on the same chromosome, and the strength of linkage disequilibrium was expressed as r^2^. To assess the level of linkage disequilibrium in the 20 populations, genome-wide SNPs were applied to map the attenuation of the different populations. The GTD and BJD populations had lower levels of LD (r^2^ values) than the TKD and QD population groups (Figure 16).

## 4. Discussion

The technology of resequencing sequencing contributes significantly to the investigation of the genetic information of a vast array of species, particularly non-model organisms [5,6,7,8]. Through flow karyotyping, which detects alterations in chromosome number and structure, we analyzed chromosomal polymorphisms [83,84]. SNP and InDel mutation rates can be accelerated by the polyploidy of plant chromosomes under unfavorable conditions, which can hinder the detection and analysis of these genetic variants within the genome [37,85,86]. As a result, flow cytometric karyotype analysis is of the utmost importance in plant genomics, which provides essential information for subsequent genome sequencing, SNP detection, and genome assembly by facilitating the prediction of the number and structure of chromosomal variants [87,88]. Genomic DNA sequences frequently comprise an extensive number of SNPs and InDels, which can be efficiently detected and exhaustively examined through the utilization of high-depth resequencing technology [7]. Subsequent information analysis made use of sample base error rates, base type distribution checks, maps showing the depth distribution of sample chromosome coverage, statistics on the distribution of insertion fragments, and sample depth distribution posts. Moreover, the assessment of GC content holds significance as it is considered a characteristic feature in genome organization [89]. The customary spectrum of GC content in eukaryotic genomes extends from 30% to 65% [90]. This study’s GC concentrations fell well within this range, indicating that the sequencing data were accurate [91].

When PIC ≥ 0.5, the locus is considered highly polymorphic. For 0.25 ≤ PIC < 0.5, the locus is moderately polymorphic, while a PIC < 0.25 indicates low polymorphism. Based on our results, the genetic diversity observed in *Z. loczyi* falls within the range of 0.25 ≤ PIC < 0.5, indicating moderate genetic diversity. Adaptive genetic variation is influenced by various factors such as geology, climate, and altitude [92]. The values of He and Ho were lower in the GTD region than in the other three regions. We hypothesize that natural selection will likely favorably select environmentally acclimated individuals, thereby causing a shift in the genotypic distribution. Particular deviations from Harry Weinberg may result from this, particularly in cases where particular genotypes possess a substantial fitness advantage or disadvantage. Nevertheless, the possibility remains that additional factors, such as genetic drift, migration, gene interactions, natural selection, and so forth, could exert an influence. These results suggest that regions with lower genetic differentiation among populations exhibit higher genetic variation [93]. Furthermore, comparing genetic diversity among populations also emphasizes the importance of genetic conservation efforts for *Z. loczyi*. An interesting result is that the QD group has the highest Nei’s diversity index, while the GTD group has the lowest. Genetic diversity is an important indicator of a population’s ability to adapt to changing environments and potential threats [94]. A higher Nei’s diversity index in the QD group implies that this population may possess a wider range of genetic variation, which could potentially provide them with a greater capacity to respond to selective pressures or environmental changes. On the other hand, the lower Nei’s diversity index observed in the GTD group indicates that this population has less genetic variation [28]. This could imply a reduced ability to adapt to environmental challenges due to this limited gene pool [95,96].

There is substantial evidence to suggest that the four genetic categories closely align with regions of geographical distribution. The population structure is in accordance with the species’ arid evolution [21]. The overlap between the population structure of K = 4 and geographic partitioning supports a genetic–geographic boundary correspondence [97]. This implies that *Z. loczyi* has evolved to differentiate advantageously due to variations in the natural environment and geography across the four sampling regions. In particular, probable gene flow between the BJD and QD populations was observed. This hypothetical scenario posits that although the four primary deserts exhibit conspicuous distinctions, there remains potential for genetic material exchange and interconnection among specific desert populations. Principal component analyses and our phylogenetic tree indicate that there may be some gene flow between BJD and QD [32]. Reduced genetic associations between the analyzed SNPs were indicated by the lower LD in the GTD and BJD populations, which suggested the possibility of recombination events and increased genetic diversity in these populations [98]. On the other hand, the larger LD values observed in the TKD and QD population groups suggest a more robust genetic association and increased correlation among the analyzed SNPs. This suggests that certain genomic regions may be undergoing selection or genetic linkage [99,100]. Nevertheless, gene migration represents merely one among several possible explanations [101]. Incomplete germline classification, convergent evolution, the structure of ancestral populations, and additional variables may also account for our results [102,103,104,105].

Over two million square kilometers in northern China are classified as sandy and/or desolate terrain [106]. Variations in the distribution of plant species among the four primary deserts are discernible within the ancient genus *Zygophyllum* [46]. Recurrent climatic fluctuations throughout the Quaternary Ice Age may have prompted plant species to seek sanctuary in regions more conducive to survival during cooler periods [107,108]. After the Ice Age, certain plant species migrated and disseminated from their refuges to other regions [1,109]. TKD in the Tarim Basin began to appear during the mid-Pleistocene (0.78–0.13 Mya) as a product of the fourth uplift of the Tibetan Plateau (3.5–1.6 Mya) [110]. By the Holocene the desert was in a phase of major expansion [34]. Therefore, during the late Pleistocene (0.13–0.01 Mya) LGM period, many large lakes and marshes existed in the TKD [111]. Furthermore, we hypothesize that *Z. loczyi* may have sought refuge in the Tarim Basin. As a result of subsequent environmental degradation in TKD, *Z. loczyi* populations gradually migrated northward and expanded into GTD [112]. Hexi Corridor wind-sand landforms emerged during the transition from the Late Pleistocene to the Holocene [111]. The subsequent developments might have played a role in the dispersal and migration of *Z. loczyi* populations to BJD and QD. At this time, the BJD region was not blanketed by glaciers. QD underwent an upward trend throughout the Tertiary Himalayan orogeny [113]. The onset of arid tropical vegetation is composed primarily of plant species indigenous to the southern littoral of the Paleo-Mediterranean area [114]. Current distribution patterns may be the result of the events described above, with the QDs retaining the greatest genetic diversity.

In summary, our research provides significant contributions to the understanding of the ecological differentiation and population genetics of *Z. loczyi* populations in China. Some of these results are applicable to conservation initiatives on a practical level, and they lay the groundwork for further investigations in fields including functional genomics, ecological genetics, and population modeling. Pursuing these directions will deepen our understanding of *Zygophyllum* and inform its conservation and sustainable management. Further studies could use SSR, cpDNA, and ITS to explore historical changes in local *Z. loczyi* populations. A deeper comprehension of the origin and evolution of desert ecosystems will result from this sequence of research efforts, which will also aid in the validation of the theory attributing to desert origins.

## 5. Conclusions

In conclusion, the resequencing of the entire genome of *Z. loczyi* at the chromosome level is presented. Population studies based on whole-genome resequencing identified three distinct genetic lineages dispersed throughout the TKD, GTD, BDJ, and QD, indicating the adaptive evolution of the species. Additionally, gene flow may occur within QD and, respectively, between the populations of TKD and BJD. Phylogenetic tree and PCA analyses indicate that the four major deserts are clearly divided, with possible causes including climate fluctuations promoted by the uplift movement of the Tibetan Plateau. The segregation of formerly dispersed desert origins of divergence is supported by our data; therefore, we hypothesize that *Z. loczyi* populations spread from one branch of the TKD to the GTD and the other branch from the TKD to the QD, which then spreads to the BJD. Understanding the implications of this paper’s discovery is crucial for the preservation of other drought-tolerant desert vegetation in Northwest China and the surrounding region.

## Figures and Tables

**Table 1:** Population information of *Z. loczyi*.

Area	Pop	Latitude	Longitude	Amount
TKD	a1	80.912446	41.430356	8
	a2	77.35496	37.60674	13
	a3	77.67286	37.79617	3
	a4	78.256189	37.509027	4
GTD	b1	88.797772	44.94489	12
	b2	89.472407	44.771408	7
	b3	89.972732	44.607383	5
	b4	83.328662	44.574587	11
QD	c1	97.233058	37.124617	6
	c2	95.60336	37.458975	11
	c3	97.334042	37.141595	15
	c4	95.377427	37.572502	8
	c5	95.287567	37.88953	10
	c6	91.039958	38.098013	14
BJD	d1	100.559867	39.710435	5
	d2	100.802402	39.587463	9
	d3	98.796332	39.895022	3
	d4	101.515437	39.19226	3
	d5	103.137744	41.685669	17
	d6	102.925948	38.442503	10

**Table 2:** Genetic diversity of the four deserts.

Group	MAF	Ae	He	Nei	Poly Marker	Ao	Ho	PIC	I
TKD	0.27	1.000–2.000	0.035–0.500	0.036–0.533	45,656	1.000–2.000	0.036–1.000	0.034–0.375	0.090–0.693
		(1.441)	(0.353)	(0.360)		(1.718)	(0.414)	(0.280)	(0.523)
GTD	0.25	1.000–2.000	0.028–0.500	0.029–0.517	42,241	1.000–2.000	0.029–1.000	0.028–0.375	0.075–0.693
		(1.370)	(0.318)	(0.323)		(1.664)	(0.388)	(0.253)	(0.476)
QD	0.28	1.000–2.000	0.015–0.500	0.015–0.507	50,167	1.000–2.000	0.015–1.000	0.015–0.375	0.044–0.693
		(1.500)	(0.365)	(0.368)		(1.789)	(0.362)	(0.290)	(0.540)
BJD	0.26	1.000–2.000	0.025–0.500	0.026–0.516	41,169	1.000–2.000	0.026–1.000	0.025–0.375	0.069–0.69
		(1.375)	(0.333)	(0.337)		(1.647)	(0.404)	(0.265)	(0.498)

(MAF = average MAF, Ae = expected allele number, He = expected heterozygous number, Nei = Nei diversity index, Mp = number of poly markers, Ao = observed allele number, Ho = observed heterozygous number, PIC = Polymorphism information content, I = Shannon–Wiener index).

## Data Availability

The data presented in this study are available on request from the corresponding author.

## Supplementary Materials

The following supporting information can be downloaded at: https://www.mdpi.com/article/10.3390/genes14122152/s1, Table S1: Statistical information for each sample; *Zygophyllum loczyi* map depth; *Zygophyllum loczyi* raw.
